# Acute Cauda Equina Syndrome With Clinicoradiological Discordance Treated via Unilateral Biportal Endoscopy: A Case Report

**DOI:** 10.1155/crnm/9001940

**Published:** 2026-09-12

**Authors:** Qiudong Wu, Yujian Zhang, Jiahao Han, Weidong Guo, Bo Liao

**Affiliations:** ^1^ School of Medicine, Northwest University, Xi’an 710069, China, nwu.edu.cn; ^2^ Department of Orthopedics, Advance Spine Surgery Center, Tangdu Hospital, Fourth Military Medical University, Xi’an 710038, China, fmmu.edu.cn

**Keywords:** case report, cauda equina syndrome, efficacy, lumbar disc herniation, timing of surgery, unilateral biportal endoscopy

## Abstract

Cauda equina syndrome (CES) is a severe neurosurgical emergency typically caused by lumbar disc herniation. Its diverse clinical manifestations include radiating lower limb pain, numbness, decreased muscle strength, saddle anesthesia or reduced perineal sensation, bladder and bowel dysfunction (such as urinary retention and constipation), and sexual dysfunction. Early diagnosis and timely decompression surgery are crucial for improving prognosis. Currently, literature reporting the use of unilateral biportal endoscopy (UBE) for treating radiologically atypical CES remains scarce. We report a 21‐year‐old male patient who presented with the sudden onset of left lower extremity pain and numbness resulting in an antalgic posture, accompanied by acute urinary retention and constipation. Physical examination confirmed cauda equina syndrome with retention (CES‐R). Although lumbar magnetic resonance imaging (MRI) revealed a paracentral recess lesion with less central canal compromise than expected for the severity of the symptoms (Schizas Grade A), the patient underwent urgent UBE surgery due to the acute clinical presentation. Postoperatively, the antalgic posture resolved immediately, and the urinary retention and constipation completely resolved within 24 h. Six months postoperatively, the patient demonstrated complete neurological recovery and significantly improved quality of life. Ultimately, this case demonstrates that UBE represents a safe, feasible, and effective alternative option for emergency decompression in acute CES characterized by clinicoradiological discordance, allowing for thorough lateral recess exploration and motion preservation that leads to a favorable neurological recovery.

## 1. Introduction

Cauda equina syndrome (CES) is a severe neurosurgical emergency resulting from the compression of the lumbosacral nerve roots distal to the conus medullaris [1]. While etiologies include trauma, tumors, and infection, acute lumbar disc herniation (LDH) remains the primary cause, accounting for approximately 0.5%–1% of all herniation cases [2]. Clinically, CES is characterized by a constellation of symptoms including bilateral radiculopathy, lower limb weakness, saddle anesthesia, and varying degrees of pelvic organ autonomic dysfunction, such as urinary retention and constipation [3]. Because neurological recovery hinges on timely intervention, established international guidelines mandate urgent clinical evaluation and lumbar magnetic resonance imaging (MRI) whenever CES is suspected [4, 5]. Although the clinical classification of CES ranges from suspected to complete forms based on the progression of sphincter control and saddle numbness [6], surgical decompression within 24–48 h remains the gold standard to prevent irreversible ischemic neural injury [7, 8]. Classically, patients with definitive CES present with massive central LDHs that cause severe spinal canal stenosis [9]. However, paracentral disc herniations present a distinct clinical challenge; they often manifest as localized lateral recess lesions with less central canal compromise than expected on routine static axial MRI. This pronounced clinicoradiological discordance can easily lead to diagnostic hesitation, potentially delaying mandatory emergency interventions [10, 11].

The case reported involves a young patient with a paracentral LDH. Notably, despite presenting with clinical manifestations consistent with CES, the lumbar MRI findings were nontypical and rare in previous medical literature. This case highlights the distinctiveness and clinical challenges in managing CES with atypical imaging caused by paracentral disc herniation. Our case report provides diagnostic and therapeutic insights into rare CES arising from the common condition of LDH. This contributes to a deeper understanding of the etiology, treatment strategies, and prognosis of radiologically atypical CES caused by paracentral LDH.

## 2. Case Presentation

A 21‐year‐old male university student presented with a 3‐week history of radiating pain and numbness in the left lower extremity, accompanied by objective constipation for 1 week and dysuria for 1 day. He had no significant past medical history, trauma, fever, family history, allergies, or medication use. On physical examination at admission, the patient assumed an antalgic prone position due to severe radicular pain. Palpation of the spine revealed acute tenderness and local percussion pain over the L5–S1 region. The straight leg raise (SLR) test was strongly positive on the left side at 20° and negative on the right side. A structured, baseline neurological evaluation was performed at admission to confirm the diagnosis (Table 1). Based on the objective presence of painless neurogenic urinary retention, diminished sacral reflexes, bilateral saddle anesthesia, and perianal hypesthesia, the patient’s condition was definitively classified as cauda equina syndrome with retention (CES‐R).

**TABLE 1:** Baseline neurological findings at admission.

Neurological domain	Clinical findings at admission	Target spinal levels
Motor strength	Mild weakness in left ankle plantarflexion (MRC 4/5); normal strength (MRC 5/5) in knee extension, ankle dorsiflexion, and great toe extension bilaterally.	L4–S1 roots
Reflexes	Patellar reflexes were intact and symmetrical (2+); the left Achilles reflex was completely absent (0).	L2–S1 roots
Sensory status	Bilateral saddle anesthesia and perianal hypesthesia; slightly decreased sensation over the posterior left calf and sole.	L5–S4 dermatomes
Sphincter tone and reflexes	Decreased voluntary anal sphincter contraction; sluggish/diminished bulbocavernosus reflex.	S2–S4 roots
Bladder autonomic function	Painless palpable bladder distention; acute urinary retention with 400 mL drained urine upon emergency catheterization.	S2–S4 parasympathetic

Following admission, intravenous dexamethasone and mannitol were administered to alleviate pain and reduce neural edema at 14:00. Despite 4 h of conservative management, symptoms persisted, and the patient developed palpable bladder distention indicative of acute urinary retention. Emergency catheterization was performed promptly at 19:50, draining 400 mL of urine. Routine laboratory investigations were performed and within normal limits.

**FIGURE 1:**
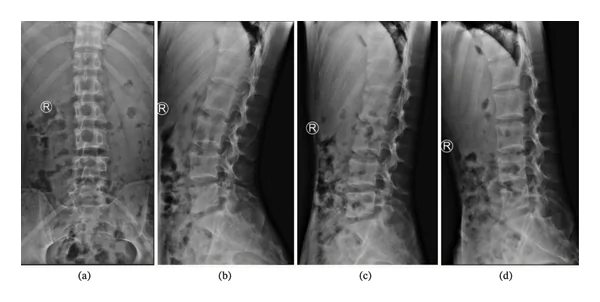
Preoperative lumbar radiographs. (a) Anteroposterior view; (b) lateral view; (c) hyperextension view; and (d) hyperflexion view.

**FIGURE 2:**
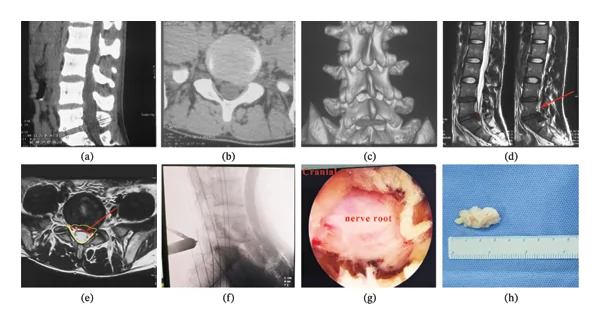
Preoperative multimodal imaging workup and intraoperative UBE decompression findings. (a–c) Preoperative lumbar computed tomography (CT) including (a) sagittal view, (b) axial view, and (c) 3D reconstruction showing intact bony alignment. (d, e) Preoperative lumbar magnetic resonance imaging (MRI) sequences. (d) Sagittal T2‐weighted images; the bidirectional red arrow (left panel) delineates the vertical craniocaudal extent of the focal canal stenosis, and the single red arrow (right panel) indicates the redundant nerve roots (RNRs). (e) Axial T2‐weighted view at the L5–S1 level; the yellow shaded region outlines the true cross‐sectional area of the spinal canal, whereas the area enclosed between the red curve and the anterior disc‐side yellow margin represents the relative intracanal space‐occupying disc mass. (f–h) Sequential intraoperative UBE landmarks: (f) fluoroscopic localization of the target level, (g) complete direct visualization of the thoroughly decompressed left S1 nerve roots, and (h) the final excised mass of the nucleus pulposus fragment.

A preoperative lumbar CT scan from an external hospital revealed a herniated disc at L5–S1 (central‐left paracentral type). Urgent lumbar spine radiography demonstrated preserved lumbar lordosis, intact vertebral bodies, and narrowing of the L5–S1 intervertebral space. Subsequent lumbar MRI confirmed a left‐sided, posteriorly extruded disc at L5–S1, causing compression of the thecal sac and the left nerve root, occlusion of the left lateral recess, and stenosis of the spinal canal at the same level (Figures 1 and 2). Quantitatively, objective grading of the canal compromise according to the Schizas classification system revealed Grade A stenosis; the central spinal canal cross‐sectional area (CSA) was well preserved with an abundance of cerebrospinal fluid and a patent central dural sac, exhibiting a canal occlusion of less than 20% [12]. The focal, space‐occupying pathology was strictly sequestered within the left paracentral region and the lateral recess, leaving the remainder of the central canal anatomically patent. Although this radiographic stenosis appeared moderate relative to the severe clinical presentation—characterized by the acute onset of urinary retention, saddle anesthesia, and radicular pain—a diagnosis of CES was strongly supported.

Given the diagnosis of CES, the patient underwent emergency unilateral biportal endoscopy (UBE) decompression, discectomy, and nerve root release under general anesthesia 8 h after admission (0:10 a.m. the following day) (Figure 2). The patient was positioned prone. Two 1‐cm incisions were made: one for the viewing portal (1.5 cm superior to the medial border of the left L5 pedicle) and one for the working portal (1.5 cm inferior to the disc space). Following blunt dissection of soft tissues and paraspinal muscles, the endoscope was inserted cephalad while instruments were advanced caudally. Under continuous saline irrigation, the endoscope and instruments were maneuvered to the surgical site adjacent to the intervertebral disc. The surgeon manipulated a 30° oblique endoscope with one hand while exposing the inferior margin of the L5 lamina with instruments in the other. After drilling and grinding the lamina margin, rongeurs removed part of the inferior lamina margin and the left ligamentum flavum. The dura mater was retracted to the contralateral side, revealing the protruding nucleus pulposus. Following removal of the herniated fragment, the S1 nerve root was explored, confirming thorough decompression and restoration of mobility in both the axillary and shoulder regions. The dural sac exhibited normal pulsation, confirming adequate canal clearance. Hemostasis was achieved using bipolar radiofrequency coagulation, and a drain was placed. Intraoperative blood loss was approximately 30 mL.

**FIGURE 3:**
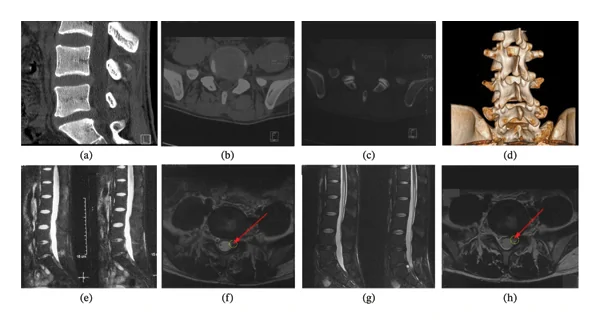
Postoperative follow‐up: (a–d) lumbar spine CT captured at 1 week postoperatively, consisting of (a) sagittal view, (b) axial soft‐tissue window, (c) axial bone window, and (d) 3D reconstruction showing optimal surgical entry and intact facet joint stability. (e, f) Lumbar spine MRI at 1 week postoperatively, displaying (e) sagittal and (f) axial views. (g, h) Lumbar spine MRI at 1 month postoperatively, displaying (g) sagittal and (h) axial views. Notably, on both the 1‐week (f) and 1‐month (h) postoperative axial sequences, the yellow circles and red arrows delineate the thoroughly decompressed nerve roots and the completely restored patency of the lateral recess.

**FIGURE 4:**
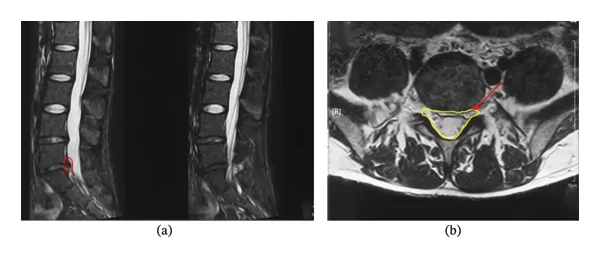
Long‐term postoperative follow‐up MRI at 6 months. (a) Sagittal fat‐suppressed T2‐weighted sequence; the red circle highlights the completely restored patency and vertical continuity of the central spinal canal at the L5–S1 index level. (b) Axial fat‐suppressed T2‐weighted sequence; the total cross‐sectional area of the spinal canal is traced/outlined, and the red arrow indicates the thoroughly decompressed nerve root with an anatomically normalized dural sac profile and clear perineural padding.

Postoperatively, the patient experienced significant improvement in left lower back pain and was able to maintain an upright posture. Urinary retention resolved within 24 h, allowing spontaneous voiding. Sensory function in the saddle area recovered by day 3 postoperatively, with bowel and bladder function returning to normal. Follow‐up lumbar MRI demonstrated marked resolution of compression. The rehabilitation strategies included physical therapy components such as lumbar strengthening exercises, bladder training, and functional training. Written informed consent was obtained from the patient himself prior to publication. The manuscript has been prepared in accordance with the CARE guidelines for case reports, while fully protecting patient privacy in compliance with ICMJE recommendations. A follow‐up lumbar MRI at 1 week postoperatively showed no spinal canal stenosis at the L5–S1 level, mild edema, and no abnormalities in cauda equina signal intensity. A follow‐up CT scan with 3D reconstruction at 1 week postoperatively revealed no significant stenosis at the L5–S1 level and good stability of the lumbar facet joints. One month postoperatively, the patient recovered well. Follow‐up lumbar MRI showed resolution of the L5–S1 disc protrusion, disappearance of edema, and no significant compression (Figure 3). At the final follow‐up (6 months), the patient reported no lumbar discomfort, complete resolution of left lower limb pain, no numbness, normal bladder and bowel function, and full resumption of normal academic and daily activities. Concurrently, the leg visual analog scale (VAS) score decreased from 8 preoperatively to 0, and the Oswestry disability index (ODI) score was reduced from 80% to 10%. Crucially, a repeat lumbar MRI performed at this 6‐month milestone demonstrated complete resolution of the previous spinal canal compression alongside an anatomically normal dural sac alignment (Figure 4). A detailed chronological timeline of the patient’s clinical course and follow‐up milestones is presented in Table 2.

**TABLE 2:** Timeline of clinical course, diagnostic workup, and therapeutic interventions (CARE guidelines).

Time point/Phase	Clinical presentation and status	Interventions, evaluations, and outcomes
Prodromal phase (3 weeks–1 day preadmission)	• Left lower extremity radicular pain and numbness (3 weeks). • Onset of objective constipation (1 week) and dysuria (1 day).	None (self‐management).

Emergency Admission (Day 1, 14:00–19:50)	• Hyperacute progression to profound bilateral saddle anesthesia. • Painless palpable bladder distention indicating acute urinary retention.	• Medical therapy: IV dexamethasone and mannitol. • Intervention: Emergency urethral catheterization (400 mL drained). • Lab work: Routine blood tests (all within normal limits).

Diagnostic and Surgical Window (Day 1 Evening–Day 2, 0:10)	• Clinical features confirming acute cauda equina syndrome (CES‐R). • Patient locked in an antalgic prone position.	•Urgent imaging: Lumbar spine radiography, CT, and MRI confirming L5–S1 paracentral disc extrusion causing selective lateral recess occlusion (Schizas Grade A central canal). • Emergency surgery: Emergent UBE decompression and discectomy (8 h postadmission).

Early postoperative period (Postoperative 24 h–Day 3)	• Antalgic posture and back pain resolved immediately. • Bladder retention resolved within 24 h with normal voiding. • Full return of saddle sensation and bowel function by Day 3.	• Postoperative monitoring and drain removal (blood loss: ∼30 mL). • Initiation of early rehabilitation strategies (lumbar exercises and bladder training).

Short‐term follow‐up (1 week–1 month)	Complete resolution of radiating leg pain and numbness; stable physical status.	• 1‐week imaging: MRI and 3D‐CT showed complete decompression at L5‐S1 and good facet joint stability. • 1‐month imaging: MRI confirmed disappearance of local neural edema and disc protrusion.

Final follow‐up (6 months)	Complete neurological recovery with full resumption of normal daily and academic activities; normal pelvic organ function maintained.	•Final comprehensive clinical evaluation. • Leg pain VAS score decreased from 8 to 0; ODI score reduced from 80% to 10%. • Follow‐up imaging: Repeat lumbar MRI at 6 months postoperatively demonstrated complete resolution of the previous spinal canal compression and an anatomically normal dural sac alignment.

## 3. Discussion

In this report, we present a rare and diagnostically challenging case of acute CES caused by an L5–S1 paracentral disc herniation. The case is characterized by a significant clinicoradiological discordance, wherein routine axial sequences demonstrated a patent central dural sac despite frank CES symptoms such as acute urinary retention and saddle anesthesia. Classically, complete bilateral CES is precipitated by a massive central disc extrusion causing a critical canal block exceeding 50% [13]. Conversely, our case demonstrates that severe sacral root deficits can be provoked by a localized, paracentral fragment rather than global canal obliteration. The successful clinical outcome in this case reinforces the fundamental neurosurgical axiom that clinical presentation must supersede moderate imaging findings in emergency decision‐making; furthermore, it demonstrates that UBE can safely and rapidly achieve thorough decompression once the emergency surgical indication is established.

To secure a precise diagnosis of acute CES, alternative etiologies were systematically evaluated and excluded based on established clinical and diagnostic frameworks [3]. First, conus medullaris syndrome was ruled out by the absence of upper motor neuron signs, as evidenced by intact, symmetrical patellar reflexes and bilateral negative Babinski signs. Second, inflammatory, infectious, or neoplastic intradural pathologies were discounted; the patient was afebrile, baseline inflammatory markers (white blood cell count, C‐reactive protein, and erythrocyte sedimentation rate) were within normal ranges, and sagittal T2‐weighted MRI demonstrated unobstructed cerebrospinal fluid signals without intradural masses or leptomeningeal enhancement. Finally, functional urinary retention secondary to severe spinal pain was excluded; unlike a pain‐induced functional overlay, the patient’s urinary retention was entirely painless and accompanied by objective neurological deficits, including bilateral sacral (S2–S4) saddle anesthesia and diminished voluntary anal sphincter tone. This comprehensive differential analysis strongly supports that the acute pelvic organ autonomic dysfunction was primarily driven by the focal mechanical compression at the L5–S1 level [14].

The mismatch between imaging severity and clinical presentation poses a critical diagnostic challenge in CES management. Atypical or seemingly reassuring imaging frequently contributes to surgical hesitation, potentially delaying emergency decompression and adversely affecting neurological outcomes [15]. While most contemporary studies focus on consistent cases with massive central herniation [13], our case aligns with less common, discordant presentations where severe symptoms coexist with radiographic findings [16, 17]. What distinctly distinguishes this case is the hyperurgent presentation of complete sacral nerve root deficits (CES‐R) driven by a well‐localized paracentral extrusion rather than a central block, a reality closely tied to the prolapse‐to‐canal ratio [18]. Unlike migrated fragments that often cause symptoms disproportionate to their apparent size [19, 20], this well‐contained paracentral fragment selectively entrapped the S2–S4 pathways within the lateral recess. This demonstrates that profound autonomic impairment can manifest rapidly even when static axial imaging shows a patent central sac, failing to capture dynamic, position‐dependent shifts of the cauda equina within the subarachnoid space [21, 22]. Therefore, the presence of objective clinical red flags warrants immediate intervention regardless of radiological severity.

Unlike the asymptomatic radiological compression described by Soleiman [23], our case presents the inverse challenge: severe symptoms with radiologically subtle pathology on routine MRI assessment. Crucially, the “stenosis” observed in this acute setting represents a relative, focal neural occlusion caused strictly by the extruded disc fragment rather than a pre‐existing chronic degenerative bony stenosis. First, although the central canal appeared patent on axial sequences, sagittal MRI demonstrated a redundant nerve root (RNR) phenomenon with tortuosity of the left‐sided nerve roots [24]. While RNRs typically reflect chronic canal stenosis, in this acute setting, it likely represents acute root crowding or an underlying anatomical predisposition. Second, because routine axial MRI sequences focus tightly on the indexed disc space, the inferior caudal extension of the extruded fragment may not be fully captured, leading to an underestimation of the true neural compromise. Third, localized microvascular pressure on radicular vessels can cause ischemic deficits disproportionate to the observed mass effect [16]. Finally, supine MRI inherently minimizes intradiscal pressure compared with upright or antalgic postures [25]. Furthermore, focal selective involvement of the S2–S4 roots within the lateral recess can disrupt the efferent parasympathetic pathways via the pelvic splanchnic nerves. This selective neurological dampening leads to detrusor underactivity (manifesting as difficulty initiating urination) and a concomitant loss of colorectal peristalsis, providing a plausible anatomical explanation for the co‐occurrence of acute neurogenic constipation and dysuria without requiring global dural sac compression [13].

In this clinical scenario, urgent surgical intervention serves a dual role as both diagnostic confirmation and definitive treatment. Regarding surgical decision‐making, while traditional open laminectomy remains the established gold standard for acute CES, it necessitates extensive paraspinal muscle stripping and the disruption of midline ligamentous complexes, which can compromise long‐term biomechanical stability [26]. Given that our patient was a 21‐year‐old university student with a structurally well‐developed, stable spine and an isolated paracentral herniation, a motion‐preserving, tissue‐sparing approach was highly favored. While UBE is routinely utilized for elective lumbar disorders in our center, its deployment in hyperacute spinal emergencies like CES is frequently cautioned against due to potential learning‐curve delays. However, our surgical team’s extensive proficiency (> 500 advanced endoscopic spine cases) counteracted this limitation, ensuring rapid, immediate decompression without any procedural delay relative to traditional open surgery. Intraoperatively, the pathology was approached strictly from the symptomatic left ipsilateral side. Utilizing the high‐definition, dynamic 30° oblique visualization, the team was able to look beneath the moderate axial MRI profiles and explore across the central canal [27]. This facilitated a precise unilateral laminotomy with thorough clearance of the paracentral fragment and contralateral recess exploration, completely releasing the sacral nerve roots without requiring formal contralateral bony resection or destabilizing the midline structures [28]. This targeted capability resulted in immediate symptomatic relief and sustained complete neurological recovery at the 6‐month follow‐up, supporting UBE as a feasible and safe alternative option when clinical suspicion of CES remains high despite atypical imaging findings.

## 4. Conclusion

In conclusion, this case highlights that severe acute CES can arise from a paracentral disc herniation despite a relatively preserved central canal on routine axial MRI. When marked clinicoradiological discordance exists, clinical red flags must guide emergency decision‐making. For this specific presentation, UBE represents a safe, feasible, and effective alternative surgical option for emergency decompression. It offers the dual benefits of direct, multiangle visualization of occult recess stenosis and maximal preservation of spinal motion structures, yielding excellent clinical and radiological outcomes at long‐term follow‐up.

**Nomenclature**
CES: Cauda equina syndrome
LDH: Lumbar disc herniation
UBE: Unilateral biportal endoscopy
PELD: Percutaneous endoscopic lumbar discectomy
RNR: Redundant nerve root

## Author Contributions

All authors were involved in the conception and design of this study. Jiahao Han recorded and analyzed data related to the patient’s paracentral lumbar disc herniation causing atypical imaging‐based cauda equina syndrome. Weidong Guo and Bo Liao participated in the patient’s diagnosis and treatment, conducted follow‐up care, and obtained clinical data. Qiudong Wu and Yujian Zhang were responsible for drafting and reviewing this paper.

## Funding

The authors declare that financial support was received for the research, authorship, and/or publication of this article. We acknowledge the financial support received from the National Natural Science Foundation of China Regional Innovation and Development Joint Fund Project (No. U24A20115), Xi’an Science and Technology Funds (No. 2024YXYJ0007), and Tangdu Hospital Discipline Development Initiative (2024JCRH004).

## Disclosure

All authors have read and approved the final version of the manuscript. The funding sources played no role in the clinical decision‐making, data collection, manuscript preparation, data analysis, or the decision to submit the manuscript for publication.

## Ethics Statement

The authors have nothing to report.

## Consent

Written informed consent was obtained from the patient himself for the publication of this case report and all accompanying images.

## Conflicts of Interest

The authors declare no conflicts of interest.

## Data Availability

The data supporting the findings of this case report are available from the corresponding authors upon reasonable request. All relevant patient information has been deidentified to protect patient privacy.
